# Supplementary figures and images for: Use of zebrafish to identify host responses specific to type VI secretion system mediated interbacterial antagonism
Source: PLoS Pathog. 2024 Jul 18;20(7):e1012384. doi: 10.1371/journal.ppat.1012384 (PMC11288455; doi:10.1371/journal.ppat.1012384)

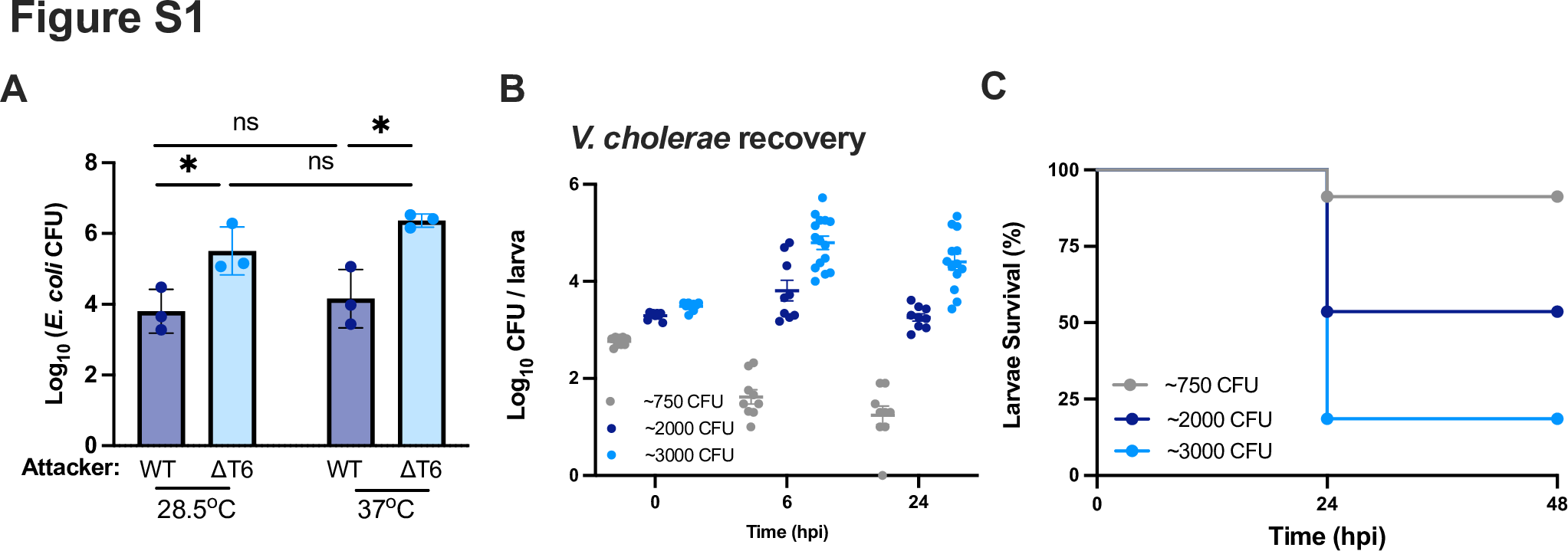

Supplement: S1 Fig — (A) E. coli recovery after in vitro competition with WT or ΔT6SS V. cholerae at 28.5°C and 37°C. Three biological replicates were performed. Significance was assessed by unpaired t-test performed on Log10 transformed values Bars represent mean ± SEM. *p<0.05 and ns (not significant). (B) Enumeration of recovered V. cholerae at 0, 6, or 24 hpi from zebrafish larvae infected with ~750 CFU, ~2000 CFU, or ~3000 CFU of WT V. cholerae. Circles represent individual larvae. Data were pooled from three independent experiments, each with 3–5 larvae per time point. Bars represent mean ± SEM. (C) Survival curves of zebrafish larvae infected with ~750 CFU, ~2000 CFU, or ~3000 CFU of WT V. cholerae. Data were pooled from three independent experiments, each with 10–16 larvae. (TIF) [file ppat.1012384.s001.tif]

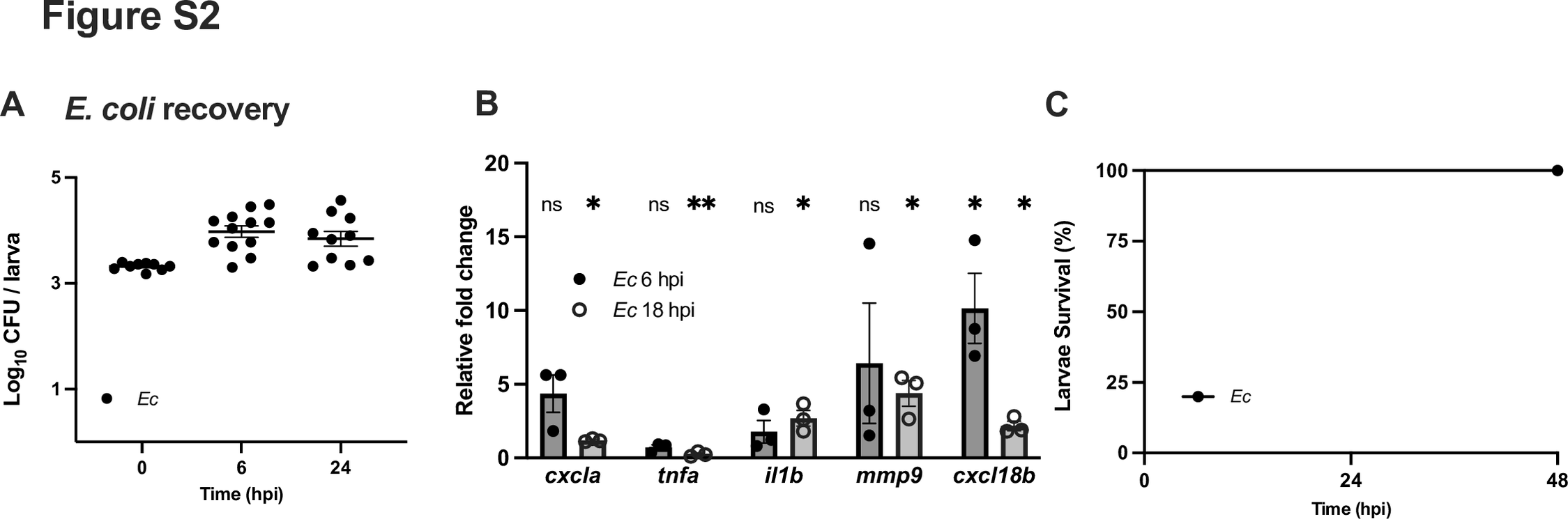

Supplement: S2 Fig — (A) Enumeration of recovered E. coli at 0, 6, or 24 hpi from zebrafish larvae infected with ~3000 CFU E. coli. Circles represent individual larvae. Data were pooled from three independent experiments, each with 4 larvae per time point. (B) Fold change in the expression of cxcl8a, tnfa, il1b, mmp9 and cxcl18b in larvae infected with ~3000 CFU E. coli relative to mock infected larvae at 6 hpi or 18 hpi. Data were pooled from three independent experiments, each with 10–14 larvae per time point. Significance was assessed by unpaired t-test performed on Log2 transformed values. (C) Survival curve of zebrafish larvae infected with ~3000 CFU E. coli. Data were pooled from three independent experiments, each with 12 larvae. For all panels, bars indicate mean ± SEM. *p < 0.05, **p < 0.001, and ns (not significant). (TIF) [file ppat.1012384.s002.tif]

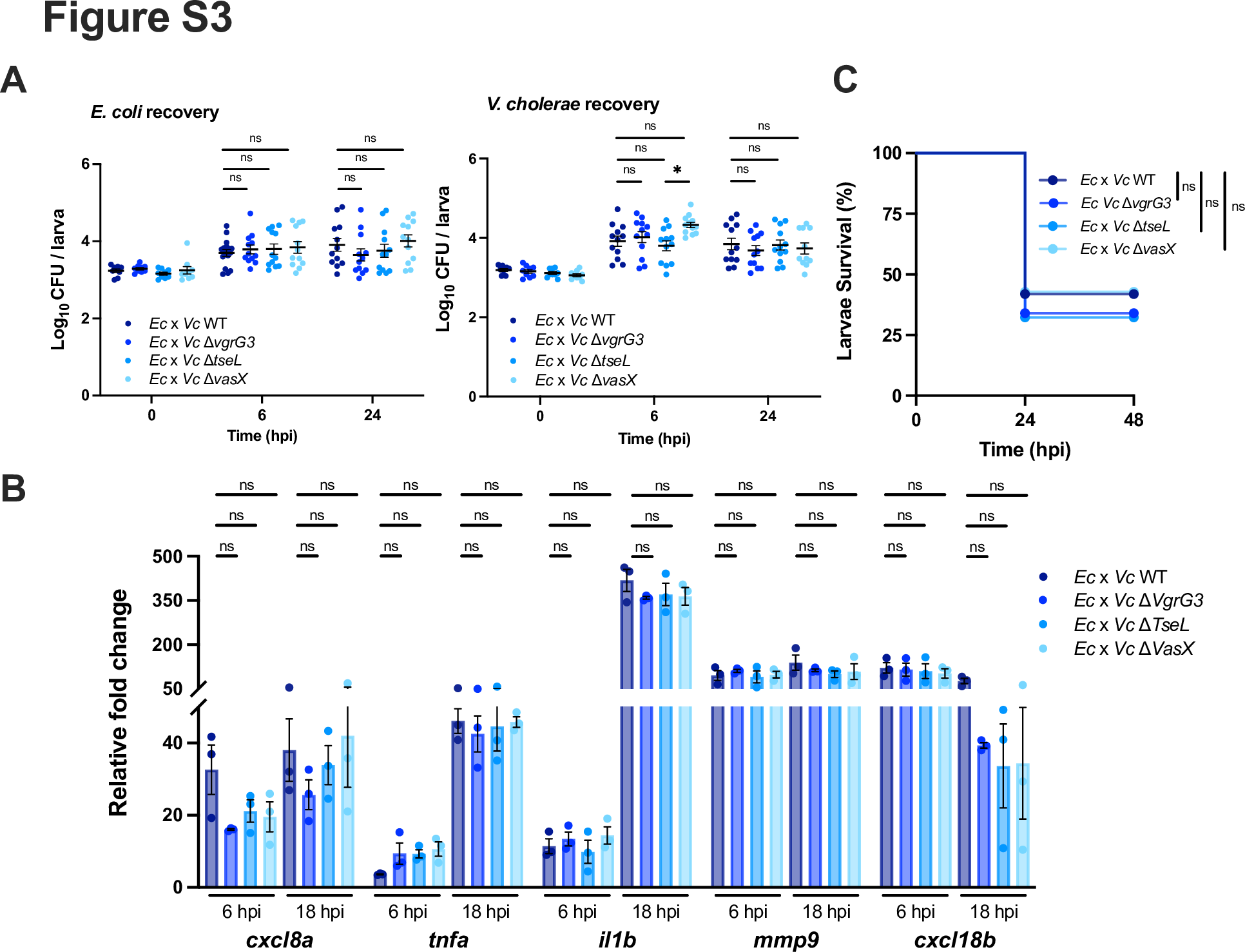

Supplement: S3 Fig — (A) Enumeration of recovered E. coli or V. cholerae at 0, 6, or 24 hpi from zebrafish larvae coinfected with a 1:1 mixture (~1500 CFU each) of E. coli and WT, ΔvgrG3, ΔtseL, or ΔvasX V. cholerae (Vc) mutants. Circles represent individual larva. Data were pooled from three independent experiments each with 3–4 larvae per time point. Significance was assessed by unpaired t-test on Log10 transformed values. (B) Fold change in the expression of cxcl8a, tnfa, il1b, mmp9 and cxcl18b in larvae coinfected with a 1:1 mixture (~1500 CFU each) of E. coli and WT, ΔvgrG3, ΔtseL, or ΔvasX V. cholerae (Vc) mutants at 6 hpi or 18 hpi. Data were pooled from three independent experiments. Significance was assessed by one-way ANOVA with Tukey’s multiple comparisons test on Log2 transformed values. (C) Survival curves of larvae coinfected with a 1:1 mixture (~1500 CFU each) of E. coli and WT, ΔvgrG3, ΔtseL, or ΔvasX V. cholerae (Vc) mutants. Data were pooled from three independent experiments, each with 15–23 larvae. Significance was assessed by log-rank Mantel-Cox test. For all panels, bars indicate mean ± SEM. *p<0.05 and ns (not significant). (TIF) [file ppat.1012384.s003.tif]

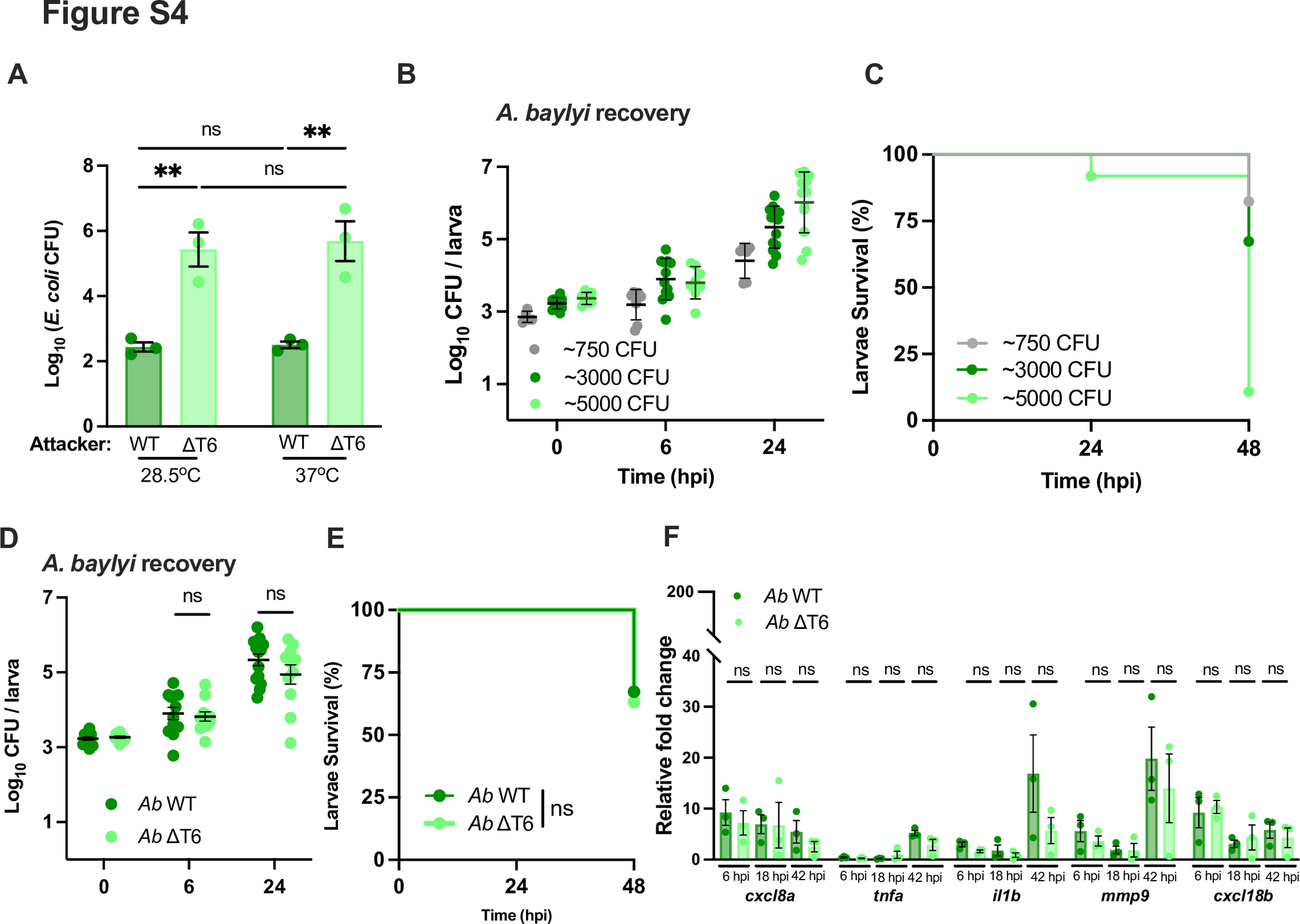

Supplement: S4 Fig — (A) E. coli recovery after in vitro competition with WT or ΔT6SS A. baylyi at 28.5°C and 37°C. Three biological replicates were performed. Significance was assessed by unpaired t-test performed on Log10 transformed values. (B) Enumeration of recovered A. baylyi at 0, 6, or 24 hpi from zebrafish larvae infected with ~750 CFU, ~3000 CFU, or ~5000 CFU of WT A. baylyi. Circles represent individual larvae. Data were pooled from three independent experiments, each with 3–5 larvae per time point. (C) Survival curves of zebrafish larvae infected with ~750 CFU, ~3000 CFU, or ~5000 CFU of WT A. baylyi. Data were pooled from three independent experiments, each with 10–19 larvae. (D) Enumeration of recovered A. baylyi (Ab) at 0, 6, or 24 hpi from larvae infected with ~2000 CFU of WT or ΔT6SS A. baylyi. Circles represent individual larvae. Data were pooled from 3 independent experiments, each with 3–4 larvae per time point. Significance was assessed using unpaired t-test on Log10-transformed values. (E) Survival curves of larvae infected with ~2000 CFU of WT or ΔT6SS A. baylyi. Data were pooled from three independent experiments, each with 15–21 larvae. Significance was assessed using log-rank Mantel-Cox test. (F) Fold change in the expression of cxcl8a, tnfa, il1b, mmp9 and cxcl18b in larvae infected with ~3000 CFU of WT or ΔT6SS A. baylyi relative to mock infected larvae at 6 hpi, 18 hpi, or 42 hpi. Data were pooled from three independent experiments, each with 10–15 larvae per time point. Significance was assessed using unpaired t-test. For all panels, bars indicate mean ± SEM. **p < 0.01, and ns (not significant). (TIF) [file ppat.1012384.s004.tif]

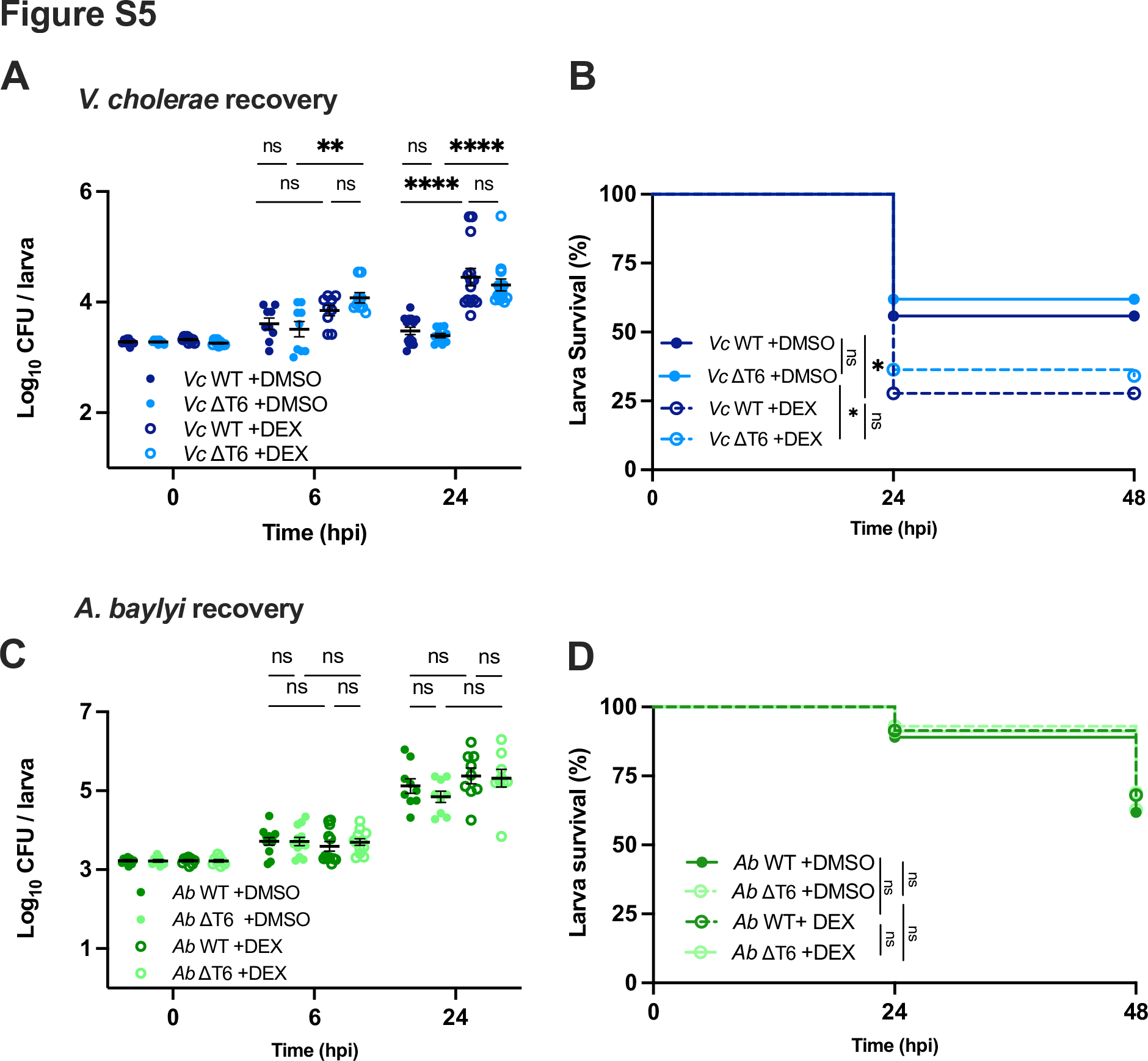

Supplement: S5 Fig — (A) Enumeration of recovered V. cholerae at 0, 6, or 24 hpi from zebrafish larvae infected with a medium dose (2000 CFU) of WT V. cholerae or ΔT6SS V. cholerae (Vc ΔT6), then immersed in 50 μg/mL dexamethasone (DEX) or solvent control (DMSO). Circles represent individual larvae. Data were pooled from three independent experiments, each with 3–5 larvae per time point. Significance was assessed by unpaired t-test on Log10 transformed values. (B) Survival curves of larvae infected with a medium dose (2000 CFU) of WT V. cholerae or ΔT6SS V. cholerae (Vc ΔT6), then immersed in 50 μg/mL DEX or DMSO. Data are pooled from three independent experiments, each with 12–18 larvae. Significance was assessed by log-rank Mantel-Cox test. (C) Enumeration of recovered A. baylyi at 0, 6, or 24 hpi from larvae infected with a medium dose (2000 CFU) of WT A. baylyi (Ab WT) or ΔT6SS A. baylyi (Ab ΔT6), then immersed in 50 μg/mL DEX or DMSO. Circles represent individual larvae. Data were pooled from three independent experiments, each with 3–4 larvae per time point. Significance was assessed by unpaired t-test on Log10 transformed values. (D) Survival curves of larvae coinfected with a medium dose (2000 CFU) of WT A. baylyi (Ab WT) or ΔT6SS A. baylyi (Ab ΔT6), then immersed in 50 μg/mL DEX or DMSO. Data are pooled from three independent experiments, each with 10–16 larvae. Significance was assessed by log-rank Mantel-Cox test. For all panels, bars indicate mean ± SEM. *p < 0.05, **p < 0.01, ***p < 0.001, ****p<0.0001, and ns (not significant). (TIF) [file ppat.1012384.s005.tif]

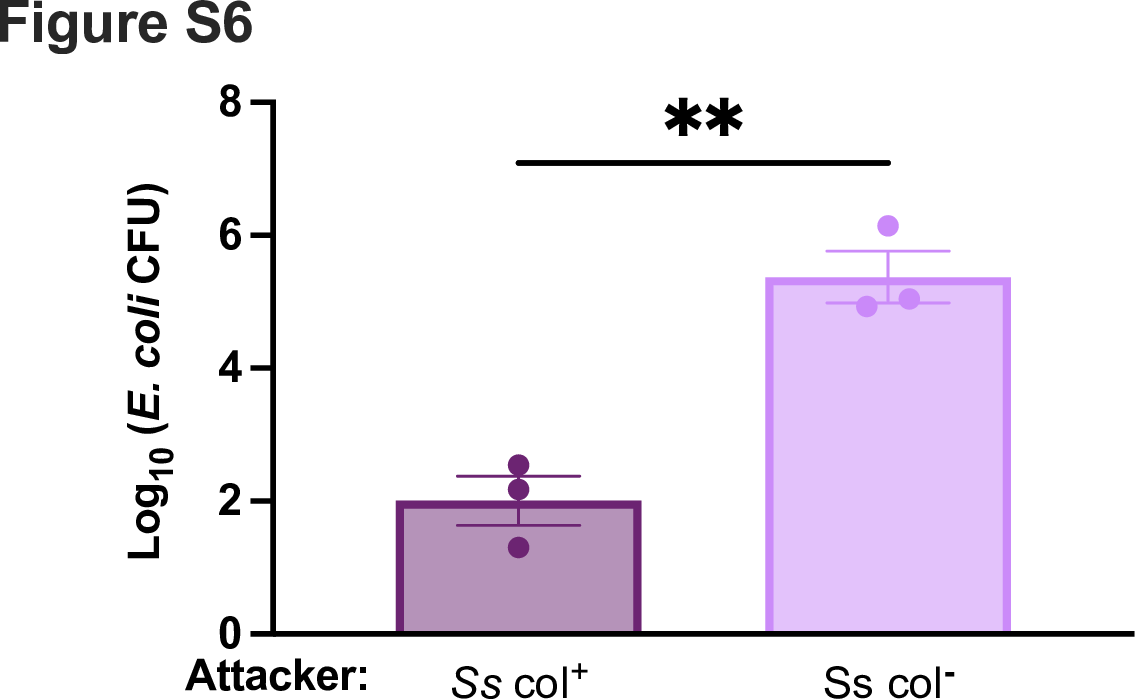

Supplement: S6 Fig — E. coli recovery after in vitro competition at 28.5°C with S. sonnei producing (col+) or not producing (col–) colicin. Three biological replicates were performed. Significance was assessed by unpaired t-test performed on Log10 transformed values. Bars represent mean ± SEM. **p<0.001. (TIF) [file ppat.1012384.s006.tif]
