# Supplementary material for: Use of zebrafish to identify host responses specific to type VI secretion system mediated interbacterial antagonism
Source: PLoS Pathog. 2024 Jul 18;20(7):e1012384. doi: 10.1371/journal.ppat.1012384 (PMC11288455; doi:10.1371/journal.ppat.1012384)
Supplement: S1 File — (DOCX) [file ppat.1012384.s007.docx]

**Supplementary file 1: Raw survival curve data (percentage survival at each time point for each experiment)**

**Figure 1: *V. cholerae* T6SS antagonism towards *E. coli* reduces host survival**

**B)**

|  | ***Vc* WT** | | | ***Vc*** $\boldsymbol{\Delta}$**T6** | | |
| --- | --- | --- | --- | --- | --- | --- |
|  | **Exp. 1 (n=12)** | **Exp. 2**  **(n=18)** | **Exp. 3**  **(n=13)** | **Exp. 1**  **(n=15)** | **Exp. 2**  **(n=17)** | **Exp. 3**  **(n=26)** |
| **0 hpi** | 100% | 100% | 100% | 100% | 100% | 100% |
| **24 hpi** | 58% | 44% | 54% | 47% | 58% | 58% |
| **48 hpi** | 58% | 44% | 54% | 47% | 58% | 58% |

**C)**

|  | ***Ec* x *Vc* WT** | | | ***Ec* x *Vc*** $\boldsymbol{\Delta}$**T6** | | |
| --- | --- | --- | --- | --- | --- | --- |
|  | **Exp. 1**  **(n=24)** | **Exp. 2**  **(n=18)** | **Exp. 3**  **(n=20)** | **Exp. 1**  **(n=19)** | **Exp. 2**  **(n=13)** | **Exp. 3**  **(n=19)** |
| **0 hpi** | 100% | 100% | 100% | 100% | 100% | 100% |
| **24 hpi** | 45.8% | 39% | 40% | 68.8% | 79% | 69% |
| **48 hpi** | 45.8% | 39% | 40% | 58% | 62% | 64% |

**Figure 3C: *A. baylyi* T6SS- mediated antagonism towards *E. coli* induces host inflammation and reduces** **host survival**

|  | ***Ec* x *Ab* WT** | | | ***Ec* x *Ab*** $\boldsymbol{\Delta}$**T6** | | |
| --- | --- | --- | --- | --- | --- | --- |
|  | **Exp. 1**  **(n=15)** | **Exp. 2**  **(n=11)** | **Exp. 3**  **(n=10)** | **Exp. 1**  **(n=14)** | **Exp. 2**  **(n=14)** | **Exp. 3**  **(n=10)** |
| **0 hpi** | 100% | 100% | 100% | 100% | 100% | 100% |
| **24 hpi** | 100% | 91% | 90% | 100% | 100% | 100% |
| **48 hpi** | 33% | 37% | 50% | 64% | 58% | 70% |

**Figure 4: Dexamethasone treatment eliminates T6SS-dependent effects on host survival**

|  | ***Ec* x *Vc* WT +DMSO** | | | | ***Ec* x *Vc*** $\boldsymbol{\Delta}$**T6 +DMSO** | | | | ***Ec* x *Vc* WT +DEX** | | | | | ***Ec* x *Vc*** $\boldsymbol{\Delta}$**T6 +DEX** | | | |
| --- | --- | --- | --- | --- | --- | --- | --- | --- | --- | --- | --- | --- | --- | --- | --- | --- | --- |
|  | **Exp. 1**  **(n=19)** | **Exp. 2**  **(n=20)** | **Exp. 3**  **(n=26)** | **Exp. 4**  **(n=20)** | **Exp. 1**  **(n=13)** | **Exp. 2**  **(n=29)** | **Exp. 3**  **(n=26)** | **Exp. 4**  **(n=15)** | **Exp. 1**  **(n=24)** | **Exp. 2**  **(n=29)** | **Exp. 3**  **(n=18)** | **Exp. 4**  **(n=18)** | **Exp. 1**  **(n=21)** | | **Exp. 2**  **(n=17)** | **Exp. 3**  **(n=15)** | **Exp. 4**  **(n=20)** |
| **0 hpi** | 100% | 100% | 100% | 100% | 100% | 100% | 100% | 100% | 100% | 100% | 100% | 100% | 100% | | 100% | 100% | 100% |
| **24 hpi** | 21% | 20% | 30% | 25% | 47% | 41% | 58% | 47% | 25% | 16% | 28% | 22.2% | 29% | | 24% | 27% | 25% |
| **48 hpi** | 21% | 20% | 30% | 25% | 47% | 41% | 58% | 47% | 25% | 16% | 28% | 22.2% | 29% | | 24% | 27% | 25% |

**C)**

|  | ***Ec* x *Ab* WT +DMSO** | | | ***Ec* x *Ab*** $\boldsymbol{\Delta}$**T6 +DMSO** | | | ***Ec* x *Ab* WT +DEX** | | | ***Ec* x *Ab*** $\boldsymbol{\Delta}$**T6 +DEX** | | |
| --- | --- | --- | --- | --- | --- | --- | --- | --- | --- | --- | --- | --- |
|  | **Exp. 1**  **(n=23)** | **Exp. 2**  **(n=16)** | **Exp. 3**  **(n=10)** | **Exp. 1**  **(n=20)** | **Exp. 2**  **(n=17)** | **Exp. 3**  **(n=10)** | **Exp. 1**  **(n=25)** | **Exp. 2**  **(n=15)** | **Exp. 3**  **(n=14)** | **Exp. 1**  **(n=18)** | **Exp. 2**  **(n=11)** | **Exp. 3**  **(n=10)** |
| **0 hpi** | 100% | 100% | 100% | 100% | 100% | 100% | 100% | 100% | 100% | 100% | 100% | 100% |
| **24 hpi** | 88% | 100% | 90% | 85% | 100% | 100% | 88% | 100% | 100% | 89% | 100% | 100% |
| **48 hpi** | 35% | 31% | 40% | 70% | 53% | 60% | 64% | 53% | 64% | 56% | 55% | 60% |

**D)**

**Figure 5A: Presence of heat-killed *E. coli* does not enhance *V. cholerae* pathogenicity in the absence of T6 mediated killing.**

|  | ***Ec* x *Vc* WT** | | | ***Ec* x *Vc*** $\boldsymbol{\Delta}$**T6** | | | **Heat-killed *Ec* x *Vc* WT** | | | **Heat-killed *Ec* x *Vc*** $\boldsymbol{\Delta}$**T6** | | |
| --- | --- | --- | --- | --- | --- | --- | --- | --- | --- | --- | --- | --- |
|  | **Exp. 1**  **(n=18)** | **Exp. 2**  **(n=16)** | **Exp. 3**  **(n=19)** | **Exp. 1**  **(n=19)** | **Exp. 2**  **(n=20)** | **Exp. 3**  **(n=11)** | **Exp. 1**  **(n=15)** | **Exp. 2**  **(n=12)** | **Exp. 3**  **(n=12)** | **Exp. 1**  **(n=17)** | **Exp. 2**  **(n=11)** | **Exp. 3**  **(n=18)** |
| **0 hpi** | 100% | 100% | 100% | 100% | 100% | 100% | 100% | 100% | 100% | 100% | 100% | 100% |
| **24 hpi** | 28% | 25% | 21% | 53% | 60% | 54.5% | 60% | 66.7% | 50% | 59% | 63.6% | 55.6% |
| **48 hpi** | 28% | 25% | 21% | 53% | 60% | 54.5% | 60% | 66.7% | 50% | 59% | 63.6% | 55.6% |

**Figure 6: Colicin-mediated antagonism does not impact host viability**

|  | ***Ec* x Col+** | | | ***Ec* x Col-** | | |
| --- | --- | --- | --- | --- | --- | --- |
|  | **Exp. 1**  **(n=14)** | **Exp. 2**  **(n=12)** | **Exp. 3**  **(n=12)** | **Exp. 1**  **(n=13)** | **Exp. 2**  **(n=13)** | **Exp. 3**  **(n=12)** |
| **0 hpi** | 100% | 100% | 100% | 100% | 100% | 100% |
| **24 hpi** | 100% | 100% | 100% | 100% | 100% | 100% |
| **48 hpi** | 100% | 100% | 100% | 100% | 100% | 100% |

**C)**

|  | ***Ec* x Col+ x *Ab*** $\boldsymbol{\Delta}$**T6** | | | ***Ec* x Col- x *Ab*** $\boldsymbol{\Delta}$**T6** | | |
| --- | --- | --- | --- | --- | --- | --- |
|  | **Exp. 1**  **(n=18)** | **Exp. 2**  **(n=25)** | **Exp. 3**  **(n=23)** | **Exp. 1**  **(n=25)** | **Exp. 2**  **(n=18)** | **Exp. 3**  **(n=17)** |
| **0 hpi** | 100% | 100% | 100% | 100% | 100% | 100% |
| **24 hpi** | 100% | 88% | 100% | 100% | 82% | 100% |
| **48 hpi** | 44.5% | 60% | 61% | 60% | 56% | 65% |

**F)**

|  | **~750 CFU** | | | **~2000 CFU** | | | **~3000 CFU** | | |
| --- | --- | --- | --- | --- | --- | --- | --- | --- | --- |
|  | **Exp. 1**  **(n=12)** | **Exp. 2**  **(n=11)** | **Exp. 3**  **(n=10)** | **Exp. 1**  **(n=15)** | **Exp. 2**  **(n=14)** | **Exp. 3**  **(n=12)** | **Exp. 1**  **(n=14)** | **Exp. 2**  **(n=16)** | **Exp. 3**  **(n=14)** |
| **0 hpi** | 100% | 100% | 100% | 100% | 100% | 100% | 100% | 100% | 100% |
| **24 hpi** | 83.3% | 100% | 100% | 46.7% | 57.2% | 58.8% | 14.2% | 12.5% | 28.6% |
| **48 hpi** | 83.3% | 100% | 100% | 46.7% | 57.2% | 58.8% | 14.2% | 12.5% | 28.6% |

**Figure S1C: Dose-dependent colonization of the zebrafish HBV by *V. cholerae***

**Figure S2C: Zebrafish larvae HBV infection with *E. coli* alone induces minimal inflammatory response**

|  | ***Ec*** | | |
| --- | --- | --- | --- |
|  | **Exp. 1**  **(n=12)** | **Exp. 2**  **(n=14)** | **Exp. 3**  **(n=10)** |
| **0 hpi** | 100% | 100% | 100% |
| **24 hpi** | 100% | 100% | 100% |
| **48 hpi** | 100% | 100% | 100% |

**Figure S3C: T6SS-dependent induction of inflammation does not require any single effector**

|  | ***Ec* x *Vc* WT** | | | ***Ec* x *Vc*** $\boldsymbol{\Delta}$**VgrG3** | | | ***Ec* x *Vc*** $\boldsymbol{\Delta}$**TseL** | | | ***Ec* x *Vc*** $\boldsymbol{\Delta}$**VasX** | | |
| --- | --- | --- | --- | --- | --- | --- | --- | --- | --- | --- | --- | --- |
|  | **Exp. 1**  **(n=15)** | **Exp. 2**  **(n=20)** | **Exp. 3**  **(n=21)** | **Exp. 1**  **(n=19)** | **Exp. 2**  **(n=22)** | **Exp. 3**  **(n=22)** | **Exp. 1**  **(n=19)** | **Exp. 2**  **(n=25)** | **Exp. 3**  **(n=15)** | **Exp. 1**  **(n=21)** | **Exp. 2**  **(n=23)** | **Exp. 3**  **(n=18)** |
| **0 hpi** | 100% | 100% | 100% | 100% | 100% | 100% | 100% | 100% | 100% | 100% | 100% | 100% |
| **24 hpi** | 47.% | 49% | 44.% | 33% | 30% | 43% | 37% | 36% | 33% | 43% | 45.5% | 36.4% |
| **48 hpi** | 47.% | 49% | 44.% | 33% | 30% | 43% | 37% | 36% | 33% | 43% | 45.5% | 36.4% |

**Figure S4: Dose-dependent colonization of the zebrafish HBV by *A. baylyi* does not require T6SS**

|  | **~750 CFU** | | | **~2000 CFU** | | | **~5000 CFU** | | |
| --- | --- | --- | --- | --- | --- | --- | --- | --- | --- |
|  | **Exp. 1**  **(n=10)** | **Exp. 2**  **(n=14)** | **Exp. 3**  **(n=10)** | **Exp. 1**  **(n=19)** | **Exp. 2**  **(n=15)** | **Exp. 3**  **(n=18)** | **Exp. 1**  **(n=10)** | **Exp. 2**  **(n=16)** | **Exp. 3**  **(n=11)** |
| **0 hpi** | 100% | 100% | 100% | 100% | 100% | 100% | 100% | 100% | 100% |
| **24 hpi** | 100% | 100% | 100% | 100% | 100% | 100% | 80% | 94% | 100% |
| **48 hpi** | 80% | 79% | 90% | 68.5% | 60% | 72% | 10% | 6% | 19% |

**C)**

|  | ***Ec* x *Ab* WT** | | | ***Ec* x *Ab*** $\boldsymbol{\Delta}$**T6** | | |
| --- | --- | --- | --- | --- | --- | --- |
|  | **Exp. 1**  **(n=21)** | **Exp. 2**  **(n=15)** | **Exp. 3**  **(n=16)** | **Exp. 1**  **(n=15)** | **Exp. 2**  **(n=12)** | **Exp. 3**  **(n=11)** |
| **0 hpi** | 100% | 100% | 100% | 100% | 100% | 100% |
| **24 hpi** | 100% | 100% | 100% | 100% | 100% | 100% |
| **48 hpi** | 77% | 77% | 69% | 77% | 58% | 64% |

**E)**

**Figure S5: Dampening the host immune response causes *V. cholerae,* but not *A. baylyi*, to become more virulent**

|  | **Vc WT +DMSO** | | | **Vc** $\boldsymbol{\Delta}$**T6 +DMSO** | | | **Vc WT +DEX** | | | | ***Vc***$\boldsymbol{\Delta}$**T6 +DEX** | | | |
| --- | --- | --- | --- | --- | --- | --- | --- | --- | --- | --- | --- | --- | --- | --- |
|  | **Exp. 1**  **(n=16)** | **Exp. 2**  **(n=11)** | **Exp. 3**  **(n=10)** | **Exp. 1**  **(n=15)** | **Exp. 2**  **(n=17)** | **Exp. 3**  **(n=12)** | **Exp. 1**  **(n=16)** | **Exp. 2**  **(n=12)** | **Exp. 3**  **(n=15)** | **Exp. 1**  **(n=14)** | | **Exp. 2**  **(n=16)** | **Exp. 3**  **(n=12)** |  |
| **0 hpi** | 100% | 100% | 100% | 100% | 100% | 100% | 100% | 100% | 100% | 100% | | 100% | 100% |  |
| **24 hpi** | 56.3% | 66.7% | 46.7% | 64.3% | 68.9% | 50% | 31.3% | 27.3% | 30% | 46.7% | | 29.4% | 33% |  |
| **48 hpi** | 56.3% | 66.7% | 46.7% | 64.3% | 68.9% | 50% | 31.3% | 27.3% | 30% | 46.7% | | 29.4% | 33% |  |

**B)**

|  | ***Ab* WT +DMSO** | | | ***Ab*** $\boldsymbol{\Delta}$**T6 +DMSO** | | | ***Ab* WT +DEX** | | | ***Ab*** $\boldsymbol{\Delta}$**T6 +DEX** | | |
| --- | --- | --- | --- | --- | --- | --- | --- | --- | --- | --- | --- | --- |
|  | **Exp. 1**  **(n=18)** | **Exp. 2**  **(n=13)** | **Exp. 3**  **(n=16)** | **Exp. 1**  **(n=18)** | **Exp. 2**  **(n=12)** | **Exp. 3**  **(n=15)** | **Exp. 1**  **(n=22)** | **Exp. 2**  **(n=14)** | **Exp. 3**  **(n=19)** | **Exp. 1**  **(n=14)** | **Exp. 2**  **(n=12)** | **Exp. 3**  **(n=17)** |
| **0 hpi** | 100% | 100% | 100% | 100% | 100% | 100% | 100% | 100% | 100% | 100% | 100% | 100% |
| **24 hpi** | 91% | 85.7% | 86.7% | 100% | 83.3% | 94.1% | 88.9% | 92.3% | 93.8% | 100% | 83.3% | 89.4% |
| **48 hpi** | 63.6% | 57.1% | 60% | 71.4% | 58.3% | 58.8% | 72% | 69.2% | 62.5% | 77.8% | 66. 7% | 63.2% |

**D)**
