# Supplementary material for: Use of zebrafish to identify host responses specific to type VI secretion system mediated interbacterial antagonism
Source: PLoS Pathog. 2024 Jul 18;20(7):e1012384. doi: 10.1371/journal.ppat.1012384 (PMC11288455; doi:10.1371/journal.ppat.1012384)
Supplement: S1 Table — (DOCX) [file ppat.1012384.s008.docx]

| S1 Table | | | | | |  |
| --- | --- | --- | --- | --- | --- | --- |
| **Organism** | **Ref in text** | **Relevant genotype** | **Plasmid** | **Relevant Features** | **Source** | |
| *V. cholerae*  2740-80 | - | Str^R^ |  | Parental strain | [40] | |
|  | *-* | Str^R^, ∆*vipA* |  | T6SS mutant of parental strain | [40] | |
|  | *Vc* WT | Str^R^, *vc1520::TnFGL3* (Kan^R^) |  | kanR inserted into *vc1520* via transposon | This study | |
|  | *Vc* ∆T6SS | Str^R^, *vc1520::TnFGL3* (Kan^R^) ∆*vipA* |  | kanR inserted into *vc1520* via transposon | This study | |
|  | *Vc* ∆VgrG3 | Str^R^, *vc1520::TnFGL3* (Kan^R^) ∆*vca0123-vca0124* |  | Deletion of VgrG3 and cognate immunity protein from “WT” | This study | |
|  | *Vc* ∆tseL | Str^R^, *vc1520::TnFGL3* (Kan^R^) ∆*vc1417-vc1421* |  | Deletion of TseL effector/immunity protein operon | This study | |
|  | *Vc* ∆vasX | Str^R^, *vc1520::TnFGL3* (Kan^R^) ∆*vca0019-vc0021* |  | Deletion of VasX effector/immunity protein operon | This study | |
| *A. baylyi*  ADP1 | *Ab* WT | Str^R^ | pMMB67EH | ADP1 transformed with pMMB67EH to confer Carb resistance | [57] | |
|  | *Ab* ∆T6SS | Str^R^, *aciad2688-aciad2694::kanR* |  | Several genes in the T6SS cluster were replaced with kanR | [57] | |
| *E. coli*  NEB10-beta | *Ec* | Str^R^ | pBAD33-mNeonGreen | Transformed with a pBAD33 to confer Cm^R^;  used for competition experiments | This study | |
| SM10 λ pir | *-* | Kan^R^, recA::RP4-2- Tc::Mu, pir | various | Conjugative donor for making Tn mutants and gene knockouts | [73] | |
| *S. sonnei*  53G | *-* | - | *-* | Parental strain | [52] | |
|  | *Ss* col*^+^* | pINV^–^ | p*rpsM-GFP* | isolate selected for loss of pINV virulence plasmid;  transformed with prpsM-GFP to confer Carb^R^ | This study | |
|  | *Ss* col*^–^* | pINV^–^, *cea::kanR-ParE* | p*rpsM-GFP* | Replacement of colicin gene (*cea*) with kanR;  transformed with prpsM-GFP to confer Carb^R^ | This study | |
