# Supplementary material for: Use of zebrafish to identify host responses specific to type VI secretion system mediated interbacterial antagonism
Source: PLoS Pathog. 2024 Jul 18;20(7):e1012384. doi: 10.1371/journal.ppat.1012384 (PMC11288455; doi:10.1371/journal.ppat.1012384)
Supplement: S2 Table — (DOCX) [file ppat.1012384.s009.docx]

| S2 Table | | | |
| --- | --- | --- | --- |
| **Primer** | **Sequence** | **Purpose** | **Source** |
| COL_DEL_F | GCAGGCTGAAACAGAACGCCAATTGAAACTGGCTGAGGATGAAGAGAAAC TCTCTACGCCGGACGCATCGTG | Amplifies *kanR-parE* cassette to replace colicin gene (*cea*) | This study |
| COL_DEL_R | GTTCCAGCAAGCACACTAAAAAGTAAAACAACAACATAACTAACTCCAGC TCACTGATCAGTGATAAGCTGTC |  |  |
| COL_EXT_F | CATTCATGCCACAGCTAAATGG | Confirmation primers for removal of colicin gene (*cea*) | This study |
| COL_EXT_R | CCTAGCGTCAGATAAGATGCTATAC |  |  |
| COL_CONF_F | AAATCGTGTCAGCCAGCA | Confirmation primers for presence of colicin plasmid | This study |
| COL_CONF_R | CTACTGAACAGAGATCCCCGTCA |  |  |
| icsB F | GGTTCCAAGATCTGGCGATTTAAGAGAATTGTAATAATC | Primers to confirm presence of pINV | [75] |
| icsB R | GGGCCTATACGCGTTGAAGATACAGAG |  |  |
| MxiG F | CTGATTGTTGGGATAAGGCTGG | Primers to confirm presence of pINV | [75] |
| MxiG R | CCGAGATCCCCTGTTTACCTC |  |  |
| ipaH1.4_F | GGGCATGAAAAAAGCTACATCC | Primers to confirm presence of pINV | [75] |
| ipaH1.4_R | CACCATTATTCGAGTACAGGGAG |  |  |
| Kan_R | TTCTATCGCCTTCTTGACGA | Primers to amplify Tn insertion site | This study |
| Vc_Degen_F | AttgtcgaatcgtaagtagcNNNNNNNNNNACGCT |  |  |
| Vc_UNIV_F | Attgtcgaatcgtaagtagc |  |  |
| VgrG3_ExtF | ATCTAGATCGCTGCGAGATCTGTATCG | Primers to confirm deletion of VgrG3/tsiV3 | This study |
| VgrG3_IntR | TCCGCGTACGACGAGGGAT |  |  |
| VgrG3_IntF | GTCTTCGCCATCATGGTGGAG |  |  |
| VgrG3_ExtR | GTCATAGTGGCTCCTCCATG |  |  |
| TseL_ExtF | ATCTAGATGAGCTACCCTTGTGAAATG | Primers to confirm deletion of TseL operon | [37] |
| TseL_IntR | CAAGCTAGGCCCACTAAGCAATAATGCGTT |  |  |
| TseL_IntF | TGCTTAGTGGGCCTAGCTTGTAATAAAGGT |  |  |
| TseL_ExtR | ATCTAGAATACGCAGAATGCTTACTCG |  |  |
| VasX_Ext_F | ATCTAGAAGTGGTGCAACGACCTACAA | Primers to confirm deletion of VasX operon | [37] |
| VasX_Int_R | TCTGAGCTACATTAATTTCCCTTGGCCTC |  |  |
| VasX_Int_F | GAAATTAATGTAGCTCAGAACTACTTACGC |  |  |
| VasX_Ext_R | ATCTAGAGCGTTGTTATTGGCGATGCT |  |  |
| *eef1a1a_*FW | AAGCTTGAAGACAACCCCAAGAGC | qRT-PCR primers for *eef1a1a* (ENSDARG00000039502) | [79] |
| *eef1a1a_*RV | ACTCCTTT AATCACTCCCACCGCA |  |  |
| *cxcl8a_*FW | TGTGTT ATTGTTTTCCTGGCATTTC | qRT-PCR primers for *cxcl8a* (ENSDARG00000104795) | [79] |
| *cxcl8a_*RV | GCGACAGCGTGGATCTACAG |  |  |
| *cxcl18b_*FW | TCTTCTGCTGCTGCTTGCG GT | qRT-PCR primers for *cxcl18b* (ENSDARG00000075045) | [79] |
| *cxcl18b_*RV | GGTGTCCCTGCGAGCACGAT |  |  |
| *Il1b_*FW | GAACAGAATGAAGCACATCAAACC | qRT-PCR primers for *il1b* (ENSDARG00000098700) | [79] |
| *Il1b_*RV | ACGGCACTGAATCCACCAC |  |  |
| *il6_*FW | TCAACTTCTCCAGCGTGATG | qRT-PCR primers for *il6* (ENSDARG00000102318) | [79] |
| *il6_*RV | TCTTTCCCTCTTTTCCTCCTG |  |  |
| *il10_*FW | CAT AACAT AAACAGTCCCTATG | qRT-PCR primers for *il10* (ENSDARG00000078147) | [79] |
| *il10_*RV | GT ACCTCTTGCATTTCACCA |  |  |
| *tnfa_*FW | AGACCTTAGACTGGAGAGATGAC | qRT-PCR primers for *tnfa* (ENSDARG00000009511) | [79] |
| *tnfa_*RV | CAAAGACACCTGGCTGTAGAC |  |  |
| *mmp9_*FW | CATTAAAGATGCCCTGATGTATCCC | qRT-PCR primers for mmp9 (ENSDARG00000042816) | [79] |
| *mmp9_*RV | AGTGGTGGTCCGTGGTTGAG |  |  |
