# Supplementary material for: Use of zebrafish to identify host responses specific to type VI secretion system mediated interbacterial antagonism
Source: PLoS Pathog. 2024 Jul 18;20(7):e1012384. doi: 10.1371/journal.ppat.1012384 (PMC11288455; doi:10.1371/journal.ppat.1012384)
Supplement: S3 Table — (DOCX) [file ppat.1012384.s010.docx]

| S3 Table |  |  |
| --- | --- | --- |
| **Plasmid** | **Function** | **Source** |
| pMMB67EH | Used to confer Carb^R^ to *A. baylyi* | [80] |
| pTNFGL3 | Mariner transposon vector used to introduce Kan^R^ into *V. cholerae* | [77] |
| pBAD33-mNeonGreen | Used to confer Cm^R^ to *E. coli* | Lab collection |
| p*rpsM-GFP* | Used to confer Carb^R^ to *S. sonnei* | [81] |
| pKD267 | Plasmid containing kanR-parE cassette | [76] |
| pKD46 | Lambda red recombineering plasmid | [76] |
| pWM91*-*ΔvgrG3 | Suicide vector for deletion of *vgrG3/tsiV3* from *V. cholerae* | [25] |
| pDS132*-*ΔvasX | Suicide vector for deletion of *vasX* operon from *V. cholerae* | [37] |
| pDS132*-*ΔtseL | Suicide vector for deletion of *tseL* operon from *V. cholerae* | [37] |
