# Supplementary material for: Use of zebrafish to identify host responses specific to type VI secretion system mediated interbacterial antagonism
Source: PLoS Pathog. 2024 Jul 18;20(7):e1012384. doi: 10.1371/journal.ppat.1012384 (PMC11288455; doi:10.1371/journal.ppat.1012384)
Supplement: Attachment — Submitted filename: Response to reviewers - FINAL.docx [file ppat.1012384.s011.docx]

Response to reviewer comments (Black are reviewer comments; Blue is our responses)

**Part I - Summary**

Reviewer #1: Killing other microbes is a widespread trait among bacterial pathogens. However, the effect of bacteria-bacteria killing on the host and the trajectory of an infection are little understood. Here, Virgo and colleagues use the zebra fish as a host model for a bacterial infection with three different bacterial pathogens and make three remarkable observations: i) T6SS-mediated killing of E. coli results in decreased host survival after infection with two pathogenic species ii) T6SS-independent killing of E. coli does not affect host survival after infection with a non-pathogenic species and iii) inhibition of the host immune system during bacterial killing has the opposite effect on host survival after infection with the one or the other pathogenic species.

In my opinion, the text is very well written, the data is presented very nicely in the figures and controls are included.

I fully support the authors' efforts in performing such in vivo experiments with all the challenges and complexity that come along with them. I think this is very much needed and the results are of broad interest to groups interested in bacterial killing and pathogenesis.

I do think though that the complexity of the model and the comparison across species requires careful interpretation of the data. The authors are probably well aware that there are a few loose ends and I do not want to request a long list of additional experiments considering the solid existing data and the journal this is submitted to. I please ask the authors to take the comments below into consideration during the revisions.

We thank the reviewer for their insightful summary and compliments. In our revision, we reinforced our findings that bacteria killed specifically by the T6SS enhances pathogenicity by demonstrating that bacterial lysate in the absence of T6SS-killed bacteria does not produce a similar effect. We have also included further controls and some additional time points to strengthen the interpretation of our results. The manuscript has been revised to include these new data and to address the reviewer critiques as outlined in this document.

Reviewer #2: Summary: The report by Virgo et al. is a creative and interesting exploration of how interbacterial dynamics alters the host inflammatory response. Although more traditional studies with mono-associations may inadvertently capture some of these dynamics, the combination of the hindbrain ventricle system and bacterial co-injections represents an innovative and useful reductive whole-organism model that will continue to be a highly useful platform for ongoing studies.

We thank the reviewer for their compliments and are pleased at their recognition of our model system as a useful tool for dissecting *in vivo* interbacterial dynamics.

Despite my overall interest and enthusiasm for this report, in my reading, I felt that several of the authors findings were either overstated, or not presented clearly enough to fully support their conclusions. Based on this, the authors should consider the addition of several experiments and controls that would enhance the strength of this report.

Overall, I believe this report merits publication, and I hope my concerns and suggestions are useful to the authors. I anticipate the editors will weigh my comments and criticisms carefully and provide advice to the authors on those which they deem most pertinent and necessary for publication within the focus of this journal.

We acknowledge that some of our conclusions were overstated. In our revised manuscript, we have included additional control experiments and have toned down our overreaching conclusions as outlined below.

**Part II – Major Issues: Key Experiments Required for Acceptance**

Reviewer #1: Consideration of alternative explanations:

1. As described below for the title, where do the authors know that differences between the findings with Vc and Ss infections are because of the different molecular mechanisms and not because of the different bacterial species used? To me, there is too much focus on the former interpretation and too little discussion of the latter. How about focusing more on the T6SS-mediated effect across the two pathogens and less on the comparison between T6SS and colicin-mediated killing in the overall story of the manuscript?

We agree that *V. cholerae* and *S. sonnei* are fundamentally different species with different infection behaviours in the host, which is why we are not actually comparing these two species. The *S. sonnei* strain we are using lacks the virulence plasmid and is strictly a colicin delivery vehicle. We have edited the text to make this clearer [lines 223-226].

Initially, we noted from the outcomes of our dexamethasone experiments (Figure 4 of the revised manuscript and newly added Figure S5) that host immune responses were suppressing *V. cholerae* infection and that T6SS-dependent effects were operating separately. Therefore, to compare T6SS to colicins, we had originally focused on *A. baylyi* where interactions between the pathogen and the innate immune response were directly connected to T6SS activity (Figure 3 of the revised manuscript). Additionally, to rule out species-dependent effects we specifically included ∆T6SS *A. baylyi* along with the colicin producer/prey (*S. sonnei*/*E. coli*) (Figure 6E,G of the revised manuscript). In our revision, to help make it clearer that we are indeed comparing T6SS killing to colicin killing in the context of the same pathogens we used to look at T6SS interactions, we have also performed the equivalent experiment with ∆T6SS *V. cholerae* (Figure 6D,F of the revised manuscript). As with our observations with *A. baylyi*, colicin-mediated killing of *E. coli* did not impact the pathogenicity of *V. cholerae*, implying that colicin-mediated killing is not a functional replacement for T6SS.

Additionally, to further address the mechanistic effects of T6SS-mediated killing on *V. cholerae* infection, we also tried to include pre-killed bacteria to confirm that having dead bacteria around is not sufficient to enable *V. cholerae* to colonize better. In addition to co-injecting *V. cholerae* with *E. coli*, we co-injected larvae with *V. cholerae* and an equivalent amount of heat-killed *E. coli*. In this case we, observed no increase *V. cholerae* colonization or innate immune stimulation (newly added Figure 5 of the revised manuscript).

These experiments show that colicin-killed cells and heat-killed lysate do not elicit the same host responses as *A. baylyi* or *V. cholerae* T6SS, nor do they result in decreased host survival, further supporting the conclusion that there is something special about T6SS-mediated bacterial killing that sensitizes the host to infection.

2. Maybe I missed this, did the authors mention the known anti-eukaryotic activity of the multiple T6SS effectors (including VgrG1) of V. cholerae? Although it seems to be as expected to the authors, to me it is unexpected that there is no phenotype of the ∆T6SS mutant on the innate immune response or host survival (l. 119ff).

We apologize for not having made it more explicit. The strain of *V. cholerae* we are using, 27040-80, carries a natural frame-shift mutation in VgrG-1 preventing its actin-crosslinking domain from being made. (Link to NCBI database entry for the VgrG-1 pseudogene:

<https://www.ncbi.nlm.nih.gov/nuccore/CP016324.1?from=1626511&to=1630001>).

Although VasX and TseL effectors do contribute to virulence against *Dictyostelium* amoeba, the magnitude of that contribution is not very high (Zheng, 2011, PMID:[21909372](https://pubmed.ncbi.nlm.nih.gov/21909372/); Miyata, 2011, PMID: [21555399](https://pubmed.ncbi.nlm.nih.gov/21555399/)). Furthermore, in experiments looking at T6SS-dependent effects of *V. cholerae* in mice, anti-eukaryotic activity, including proinflammatory cytokine expression, could be entirely ascribed to the actin-crosslinking domain of VgrG-1 (Ma, 2010, [20150509](https://pubmed.ncbi.nlm.nih.gov/20150509/)).

Even if there is no effect after infection with the V. cholerae strain alone, could this anti-eukaryotic activity still play a role after the immune system is triggered by the E. coli lysate in infections with both species?

Technically, we cannot fully rule this out. In fact, one could be tempted to speculate that the slight increase in *V. cholerae* recovery when *vasX* was knocked out despite there being no effect on *E. coli* recovery (Figure S3 in the revised manuscript) was due to some effector-specific anti-eukaryotic T6SS effect. However, if this is even a real effect, it is minor and does not have a bearing on the overall larvae host response or survival (Figures S3 B-C, 1C, 2A in the revised manuscript).

Did the authors consider injecting the zebra fish with E. coli lysate alone (corresponding to a similar number of live E. coli that were injected in the experiment), E. coli lysate together with a V. cholerae ∆T6 mutant, and E. coli lysate together with a V. cholerae WT strain? I think such experiments would very much strengthen the manuscript and help disentangle whether the observed phenotype is purely based on E. coli cell lysate, the co-occurrence of E. coli lysate in combination with V. cholerae per se (independent of the T6SS) or the co-occurrence of E. coli lysate, the pathogen V. cholerae and anti-eukaryotic activity of T6SS effectors.

We thank the reviewer for this excellent suggestion. As mentioned above, we performed coinfections of WT or ∆T6SS *V. cholerae* with either *E. coli* or an equivalent amount of heat-killed *E. coli* (newly added Figure 5 of the revised manuscript)*.* We observed that the heat-killed *E. coli* lysate did not affect larvae survival, cytokine expression, or *V. cholerae* growth with or without a functional T6SS. In fact, both conditions phenotypically matched a co-infection of ∆T6SS *V. cholerae* with *E. coli* demonstrating that T6SS-mediated bacterial killing is indeed driving our observed host responses.

Fig. 7A, I like the idea, however I think the conclusions on host health as indicated on the right of the figure are not justified with the current experimental set-up because of the use of pathogenic and non-pathogenic species for the T6SS and colicin phenotype. Further, there is no sign of inflammation shown in Figure 6, isn’t the blue line for the inflammatory response upon colin-mediated killing purely hypothetical and would require more experiments to be backed up?

The initial increase in inflammatory response was included because we felt that the intuitive default assumption would have been that injecting bacteria would result in some inflammatory response. But we agree with the reviewer that this is speculative. We have modified the figure to reflect this fact (Figure 7 in the revised manuscript). The key takeaway for the figure though, which we have hopefully now made clear, is that by 6 hours after infection, even if there was an inflammatory response, it would have returned to a baseline “no bacteria killing” level by the time virulence would be kicking in.

Additionally, the newly added 3-strain mixture experiment looking at the (non) impact of colicin-mediated killing in the presence of ∆T6SS *V. cholerae* virulence (Figure 6D,F) along with the original data showing the (non) impact of colicin-mediated killing on ∆T6SS *A. baylyi* virulence (Figure 6E,G) should fully address the reviewer’s concerns about mismatched comparisons of pathogenic to non-pathogenic species.

Reviewer #2: Major concerns:

1) Figure 2 represents foundational finding of the paper, yet the comparison between Vibrio and Vibrio + E. coli in terms of host survival and inflammatory cytokine secretion is only indirectly made through comparison of independent experiments. Although a comparison between the results of figure 1 and 2 can be made, the authors should show a direct comparison of cytokine and survival between mono-associated Vibrio and Vibrio + E. coli to clearly demonstrate the differences in host response of this key finding. Ideally this would be a discrete independent experiment, but at the very least, a compilation of data from figure 1 and 2 into a single graph (and indicated as such).

We agree and thank the reviewer for the suggestion. We have reorganized the first two Figures. Figure 1 now shows the initial foundational finding that decreased host survival and increased *V. cholerae* colonization when its T6SS has a susceptible target. Figure 2 then follows up with the cytokine expression data. The corresponding part of the results section have been modified accordingly. [lines 136-147]

2) The authors’ claim that continuous, but weaker bacterial T6SS mediated antagonism is more immunostimulatory due to its continuous and chronic release of E. coli DAMPs is a potentially reasonable conclusion but is only weakly supported by experimental evidence, and is somewhat of a logical leap as it stands now. a. Injection of colicin vs. T6SS killed E. coli into the hindbrain ventricle in the absence of Vibrio, then assess survival and inflammatory cytokine response to these forms of killed bacteria (in the absence of Vibrio or A. bayli). This would demonstrate that acute responses to a killed bacteria is the same between these two mechanisms, and not due to differential DAMP release based on mechanism of bacterial killing.

We acknowledge that we have not fully disentangled host response to dead bacteria from host response to dead bacteria in the presence of a potential pathogen. However, the suggested experiment of injecting T6SS-killed *E. coli* is not exactly doable because separating T6SS-killed *E. coli* from the T6SS+ bacteria that killed them is a practical impossibility. Similarly, it is not feasible to continuously or repeatedly administer *E. coli* lysate to the zebrafish larvae over the time scales of our experiments. However, we are not making such a strong claim. At most, we are suggesting that the presence of T6SS-killed *E. coli* is sensitizing the zebrafish larvae to infection.

With respect to the mechanistic cause of the increased immunostimulatory effect, Reviewer #1 raised a similar concern. We have devised an experiment injecting heat-killed *E. coli* lysate to address both reviewer’s comments together. As described above, we injected lysate together with *V. cholerae* (Figure 5 in the revised manuscript) and observed no differences in bacterial burden or host response compared to ∆T6SS *V. cholerae* mixed with *E. coli*, indicating that having dead bacteria alone is insufficient to generate a response. Any potential acute response that may have occurred is gone by the time we can try to detect it. Combined with the similar result for colicin-killed bacteria (Figure 6D-G of the revised manuscript), these results show that there is something unique about T6SS-killed bacteria that facilitates enhanced colonization (in the case of *V. cholerae*) or inflammation-dependent host death (in the case of *A. baylyi*).

3) The dexamethasone experiments are interesting and an excellent approach but are not properly controlled. Please include controls evaluating the effect of dexamethasone on Vibrio and A. bayli survival and inflammatory cytokine in the absence of E. coli. As it is, it is conceivable that dexamethasone treatment alone makes Vibrio infection pathogenic even in the absence of E. coli.

We thank the reviewer for the suggestion. Dexamethasone treatment generally making *V. cholerae* infection more pathogenic was precisely our interpretation. The reviewer is correct that we were missing a key control. This has now been done with the data presented in newly added supplementary Figure S5. Dexamethasone treatment had no significant effect on infection of *A. baylyi* alone, but it did increase pathogenicity of *V. cholerae* (independent of T6SS).

4) It is not clear that lethality in any of these models is due to the host response or a direct bacterial effect, nor is it particularly clear what role the host response is playing on bacterial grown. Although I believe it is beyond the scope of this report to experimentally address this question, the authors should indicate these unknowns and possibilities for addressing them in future studies.

Previous studies have demonstrated how microbial antagonism can also cause changes in expression of virulence determinants as well as drive host inflammation, implying a bacterial-antagonism dependent interplay between *V. cholerae* and the host (Zhao, 2018, PMID: [29326272](https://pubmed.ncbi.nlm.nih.gov/29326272/)). Other studies have demonstrated that *V. cholerae* T6SS-mediated antagonism towards commensals inhibits epithelial repair in the *Drosophila* model (Fast, 2020, PMID: [31995751](https://pubmed.ncbi.nlm.nih.gov/31995751/); Xu, 2024, PMID: [38340318](https://pubmed.ncbi.nlm.nih.gov/38340318/)}, again implying interplay between the host and the bacterial competition.

In the context of infection, inflammation is often seen as a key host response, central to helping clear infection (Medzhitov, 2010, PMID: [20303867](https://pubmed.ncbi.nlm.nih.gov/20303867/)). However, some pathogens, such as Salmonella Typhimurium, exploit this response to aid colonization (Galán, 2021, PMID: [34012042](https://pubmed.ncbi.nlm.nih.gov/34012042/)). In this instance, inflammation creates a niche which is more favourable to colonization, demonstrating how host response can influence bacterial growth differently depending on the pathogen in question. While the results from our dexamethasone experiments indicate that inflammation is important for managing *V. cholerae* infection (Figure 4) it also appears as though in some contexts, *V. cholerae* uses host inflammation to enhance its replication (Figure 1). Given that this is different from what we observe with *A. baylyi,* we believe that there is likely something inherent to *V. cholerae* that is also contributing towards its increase in pathogenicity in the context of host inflammation. It has been recently shown that *V. cholerae* can establish biofilm growth on the surface of immune cells as a form of predation (Vidakovic, 2023, PMID: [37295405](https://pubmed.ncbi.nlm.nih.gov/37295405/)). It is possible that *V. cholerae* has evolved a strategy of using T6SS-mediated bacterial antagonism to stimulate inflammatory responses and immune cell recruitment to facilitate this form of growth. In the future, resolving the spatial localization of T6SS-mediated killing with respect to immune cell recruitment/activity could help address this.

We have updated the manuscript to include this discussion. [lines 301-324]

**Part III – Minor Issues: Editorial and Data Presentation Modifications**

Reviewer #1:

- title: I agree with there being interbacterial competition and I agree with distinct host responses and health outcomes, I struggle with this being because of “different modes” of interbacterial competition. Are the authors sure there are no other possible explanations considering that different species were used to test the different molecular mechanisms? I suggest leaving out the first three words of the title.

Per the reviewer’s suggestion, we have modified the title to read: “Interbacterial competition elicits distinct host responses and health outcomes”

- abstract: for clarity, I suggest to add the name of the bacterial species with which colin-mediated bacterial killing was tested (as is already done for T6SS-mediated killing).

The abstract has been updated to specify that it is colicin mediated antagonism elicited by an avirulent strain of *S. sonnei.* [lines 21-24]

- for all Kaplan Meier curves with pooled data, could the authors please provide the data of the individual experiments in the supplementary data so that the consistency of the phenotype across independent experiments becomes visible?

We have now included an additional data file with the raw data values of larvae survival for each experiment (newly added Supplementary File 1). This file highlights the number of larvae used in each experiment as well as the percentage survival for each experiment and confirms that the phenotype was consistently observed across the independent experiments.

- l. 98, although this might hold, I do think more specific work would be needed to specifically conclude on “evolutionary advantages” and suggest not to do so here. Especially with an infection model that might be a very nice scientific model system but not necessarily represent the natural route for these pathogens.

We removed mention of ‘evolutionary advantages’ and instead comment that ‘these findings provide insight into the mechanisms of immune activation’ [line 101-102]

Reviewer #2:

1) Please show total n values for all survival curves. In your methods you say that each experiment represents several independent pools of 10-15 fish – while this seems appropriate, please display the actual total number of fish that comprise each survival experiment either on the graph or in the figure legend.

Reviewer #1 made a similar comment (See response to comment above). We have included an additional data file with the raw data values of larvae survival for each experiment (newly added Supplementary File 1).

2) I strong suggest changing the format of your survival curves. Since you are only showing three timepoints (0h, 24h and 48h), Kaplan-Meier curves are not necessary and make your results significantly harder to read and interpret.

In addition to being consistent with our previous work (examples: Torraca, 2019, PMID: [31830135](https://pubmed.ncbi.nlm.nih.gov/31830135/); Gomes, 2023, PMID: [37672585](https://pubmed.ncbi.nlm.nih.gov/37672585/)), Kaplan-Meier curves are standard to present larvae survival data in the zebrafish infection community (examples: Harvie, 2013, PMID: [23090960](https://pubmed.ncbi.nlm.nih.gov/23090960/); Viana, 2023, PMID: [37155695](https://pubmed.ncbi.nlm.nih.gov/37155695/)). We did consider presenting the data as bar graphs or as a line graph, but these plots were just as if not more confusing. Moreover, larvae at individual time points should not be considered in isolation from other time points, as larvae are followed over time and our statistical differences take this into account using Kaplan-Meier curves.

3) None of your experiments clearly include injection controls. Please briefly address how you controlled for off target injection-based lethality within each experiment (or include that data if you have it).

Injection controls are not typically necessary as we have a 100% larvae survival rate when performing injections with non-pathogenic material, i.e. injections with *E. coli* alone (Supplemental Figure S2) or *E. coli* with *S. sonnei* (Figure 6C). Given that our injection methodology does not change throughout our work and to minimize the use of animals, we did not feel it was necessary or appropriate to perform injection controls for each individual experiment.

4) Figure 3 is inconclusive and belongs in the supplement. If the T6SS were employed in the absence of any effector mechanism, would perhaps the same effects on host response be observed? If the authors performed a combined knockout of the three effectors, then perhaps this would merit this figure belonging in the main text. This may be beyond the scope of this report, but as it is, it only raises questions about possible overall mechanism.

We considered doing this. However, knocking out all three effectors unfortunately results in inactivation of the T6SS machinery in *V. cholerae* (Dong, 2013, [23362380](https://pubmed.ncbi.nlm.nih.gov/23362380/)). Per the reviewer’s suggestion, we have moved these data to the supplement. It is now Supplementary Figure S3.

5) In the discussion, the claims about perceived evolutionary trajectories of these microbes are overstated (line 235, 254, 261, 276) and the authors should either clarify or moderate these assertions.

These statements have all now been clarified or removed [lines: 35-36, 101-102, 253-254, 272-274, 281-284]
